# Stability of Fentanyl Citrate, Hydromorphone Hydrochloride, Ketamine Hydrochloride, Midazolam, Morphine Sulfate, and Pentobarbital Sodium in Polypropylene Syringes

**DOI:** 10.3390/pharmacy3040379

**Published:** 2015-12-16

**Authors:** Collin Anderson, Mark MacKay

**Affiliations:** Primary Children’s Hospital, 100 Mario Capecchi Dr., Salt Lake City, UT 84113, USA; E-Mail: mark.mackay@imail.org

**Keywords:** stability, pharmacy, fentanyl, hydromorphone, ketamine, midazolam, morphine, pentobarbital

## Abstract

Purpose: Determine the stability of fentanyl 10 mcg/mL in 0.9% sodium chloride, fentanyl 10 mcg/mL in 5% dextrose, fentanyl 50 mcg/mL, hydromorphone 100 mcg/mL in 0.9% sodium chloride, ketamine 10 mg/mL, midazolam 0.4 mg/mL in 5% dextrose, midazolam 5 mg/mL, morphine 1 mg/mL in 0.9% sodium chloride, morphine 1 mg/mL in 5% dextrose, and pentobarbital 50 mg/mL when stored as single drug entities at room temperature in polypropylene syringes. Methods: Four 5 mL samples of each drug and concentration were prepared in 10 mL polypropylene syringes. The samples were stored at ambient room temperature in a locked cabinet. Triplicate determinations of drug concentration for each sample were performed initially, on day 50 or 51, and on day 100 using high-performance liquid chromatography with diode-array detection. Results: With the exception of the hydromorphone 100 mcg/mL dilution, all compounds were found to contain greater than 95% of their initial concentration remaining at 100 days. Each sample remained clear and colorless when visually inspected.

## 1. Introduction

Controlled substances play an integral role in the pharmacological management of pain and sedation. Frequent use of these agents as analgesics and sedatives make it desirable for them to be readily available in convenient dosage forms to those who will be administering the drug substances. Federal and state regulations require additional human resources to be employed in the tracking of these agents. Automatic dispensing machines are nearly universally utilized by pharmacies within heath systems as a tool to meet the aforementioned purposes of ready availability and to meet security and documentation requirements set forth by regulatory agencies such as the drug enforcement agency. Providing the drug in the most appropriate dosage form for ease of use and the mitigation of medication errors is desirable. Pediatric institutions often need to compound drugs at lower concentrations than commercially available. Additionally, the volume provided commercially may be in excess of that which would be reasonably administered to certain pediatric patient populations. Waste is reduced and the risk of overdose is diminished by providing the compound in an amount closer to that which will likely be prescribed and administered to the patient. 

Five controlled substances intended for intermittent administration (fentanyl 10 mcg/mL, hydromorphone 500 mcg/5 mL, ketamine 30 mg/3 mL, morphine 2 mg/2 mL, pentobarbital 100 mg/2 mL) are compounded at our institution for placement in automatic dispensing machines located throughout the hospital. In addition, we currently compound five controlled substance drips at concentrations and in volumes suitable for a pediatric patient population (Table 1). Having controlled substance drips compounded and available from the central pharmacy narcotic station potentially reduces the risk of medication error which can occur at busy times during on demand compounding. The drips are loaded into the central narcotic station at the concentration and volume which they will ultimately be distributed to the patient, thus increasing efficiency of pharmacy operations. The drug compounds are tracked through our central narcotic station and automatic dispensing machines throughout the hospital. 

**Table 1:** Batch Compounded Controlled Substance Drips.

	Concentration	Volume (mL)
Fentanyl	10 mcg/mL	10, 30
Fentanyl	50 mcg/mL	10, 30
Midazolam	0.4 mg/mL	10, 30
Midazolam	5 mg/mL	10, 30
Morphine	1 mg/mL	10, 30

Published chemical stability studies are available for fentanyl 50 mcg/mL [1], fentanyl 5 mcg/mL [2], hydromorphone 100 mcg/mL [3], ketamine 10 mg/mL [4], midazolam 5 mg/mL [5], and morphine 1 mg/mL [6] concentrations in syringes. Yet, these referenced studies did not extend to the desired 100 day limit. At the time of publication we are unaware of chemical stability studies for fentanyl 10 mcg/mL or midazolam 0.4 mg/mL compounds.

## 2. Experimental Section

Materials. Fentanyl (fentanyl citrate inj., USP 50 mcg/mL, 2 mL, Baxter Healthcare, Deerfield, IL, lot 022371; fentanyl citrate inj., USP 50 mcg/mL, 2 mL, Hospira, Lake Forest, IL, lot 06419DK), hydromorphone (hydromorphone HCl inj., USP 2 mg/mL, 1 mL, Westward, Eatontown, NJ, lot 012366), ketamine (ketamine HCl inj., USP 10 mg/mL, 20 mL, JHP Pharmaceuticals, Rochester, MI, lot 293616), midazolam (midazolam inj., USP 5 mg/mL, 2 mL, Hospira, Lake Forest, IL, lot 11132DK), morphine (morphine sulfate inj., USP 50 mg/mL, 20 mL, Hospira, Lake Forest, IL, lot 12094DK), pentobarbital (pentobarbital sodium inj., USP 50 mg/mL, 50 mL, Akorn, Lake Forest, IL lot 080103F), 5% dextrose injection (5% Dextrose inj., USP 50 mL, Hospira, Lake Forest, IL, lot 09129JT), 0.9% sodium chloride injection (0.9% sodium chloride injection, USP, Hospira, Lake Forest, IL, lot 10311DK), syringes (Becton-Dickinson (BD) Luer-Lok Syringe, 10 mL, Franklin Lakes, NJ, Ref. No. 309605), and test tubes (Borosilicate Glass Disposable Culture Tubes, 13 × 100 mm, Kimble Chase, Vineland, NJ) were obtained commercially. HPLC grade water (water, HPLC grade, Fischer Scientific, Fair Lawn, NJ, lot 116273), acetonitrile (acetonitrile, for HPLC, Acros, NJ, lot B00M1591), triethylamine (triethylamine 99% pure, Acros, NJ, lot A0295058), and phosphoric acid (phosphoric acid, for analysis 85 wt% solution in water, Acros, NJ. Lot A0295482) were used in preparation of the HPLC mobile phase. Forced degradation studies were performed with sodium hydroxide (sodium hydroxide, 1N, Acros, NJ, lot B00L6551), hydrochloric acid (hydrochloric acid, 1N, Acros, NJ, lot B00L1741), hydrogen peroxide (hydrogen peroxide, 3%, JT Baker, Phillipsburg, NJ, lot E21H29), and heat (60 °C). Chromatographic analysis was conducted using an Agilent 1260 HPLC system with quaternary pump (1260 Infinity quaternary pump, Agilent Technologies, Santa Clara, CA, model G1311B), autosampler (1260 Infinity standard autosampler, Agilent, model G1329B), thermostatted column compartment (1260 Infinity thermostatted Column Compartment, Agilent, model G1316A), and diode-array detector (1260 Infinity diode-array detector, Agilent, model G4212B) with ChemStation software (HPLC ChemStation, version B.04.03, Agilent) and Zorbax Eclipse Plus C18, 4.6 × 100 mm, 3.5-micron column (Zorbax Eclipse Plus C18, Agilent). 

Chromatographic analysis. The concentrations of the controlled substances were quantified using HPLC stability indicating assays. The mobile phase consisted of aqueous 0.1% triethylamine adjusted to pH 2.5 with phosphoric acid, and acetonitrile. All analyses utilized isocratic elution (1 mL/min) on a Zorbax Eclipse Plus C18, 4.6 × 100 mm, 3.5-micron column and diode-array detection. The column compartment was maintained at 25 °C. Injection volumes, mobile phase ratios, and detection wavelengths for each analysis are listed in Table 2. Due to large peak size, immediately prior to analysis fentanyl 50 mcg/mL sample aliquots were diluted to 25 mcg/mL; midazolam 0.4 mg/mL sample aliquots were diluted to 40 mcg/mL; hydromorphone 100 mcg/mL, ketamine 10 mg/mL, midazolam 5 mg/mL, morphine 1 mg/mL, and pentobarbital 50 mg/mL sample aliquots were diluted to 50 mcg/mL, respectively. Fentanyl 10 mcg/mL samples were analyzed without further dilution. 

**Table 2:** Stability of Batch Compounded Controlled Substances.

Conditions/Results	Fentanyl	Fentanyl	Fentanyl	Hydro-morphone	Ketamine	Midazolam	Midazolam	Morphine	Morphine	Pento-barbital
concentration	10 mcg/mL in NS	10 mcg/mL in D5W	50 mcg/mL	100 mcg/mL in NS	10 mg/mL	5 mg/mL	0.4 mg/mL in D5W	1 mg/mL in NS	1 mg/mL in D5W	50 mg/mL
mobile phase(buffer: ACN)	75:25	75:25	65:35	65:35	85:15	70:30	70:30	97.5:2.5	97.5:2.5	65:35
detection wavelength (nm)	210	210	210	254	210	254	254	210	210	210
injection volume (µL)	12.5	12.5	5	5	5	5	5	5	5	5
retention time (min)	5.46	5.46	1.89	2.62	3.55	2.58	2.58	3.88	3.88	4.17
Degradants ^a^ (min)	0.9 (a,b)	0.9 (a,b)	0.8 (a,b)	1.3 (a,b)	1.9 (a), 2.3 (a)	1.0 (b)	1.0 (b)	3.1 (a, b, p)	3.1 (a, b, p)	ppt (a)
initial (concentration)^b^	10.6 ± 0.1 mcg/mL	10.0 ± 0.1 mcg/mL	48.3 ± 0.4 mcg/mL	98.6 ± 0.3 mcg/mL	10.1 ± 0.02 mg/mL	5.06 ± 0.06 mg/mL	0.397 ± 0.001 mg/mL	1.05 ± 0.004 mg/mL	1.02 ± 0.01 mg/mL	48.8 ± 0.8 mg/mL
week 7–8 % remaining ^b^	104.0 ± 1.9 (day 50)	101.7 ± 3.9 (day 50)	102.7 ± 3.2 (day 50)	94.2 ± 1.6 (day 50)	102.2 ± 0.4 (day 50)	97.2 ± 1.9 (day 51)	98.5 ± 0.5 (day 51)	100.9 ± 0.2 (day 50)	100.8 ± 0.6 (day 50)	99.6 ± 2.0 (day 50)
day 100 % remaining ^b^	101.7 ± 3.0	104.0 ± 3.3	98.3 ± 4.4	90.3 ± 0.7	100.5 ± 1.5	96.5 ± 2.6	96.4 ± 1.7	101.1 ± 0.3	100.9 ± 1.0	102.2 ± 2.2
Linearity	0.9992	0.9992	0.9999	0.9999	> 0.9999	0.9996	0.9996	0.9998	0.9998	0.9994
precision	0.33	0.33	0.87	0.17	0.29	0.36	0.50	0.10	0.10	0.97
intraday coefficient of variation	0.45	0.45	1.35	1.07	0.29	0.90	0.90	0.28	0.28	1.35
interday coefficient of variation	1.74	1.74	2.82	1.11	1.48	1.05	1.05	1.63	1.63	1.52

Notes: ^a^ a = acid, b = base, p = peroxide; ^b^ mean ± SD; Triplicate determinations of four samples.

Linear standard curves were constructed from dilutions of each compound. For fentanyl 10 mcg/mL samples, the standard curve consisted of fentanyl 5, 7.5, 10, 12.5, and 15 mcg/mL concentrations. Fentanyl 5, 10, 25, 40, and 50 mcg/mL concentrations were used for the standard curve corresponding to analysis of fentanyl 50 mcg/mL samples. All subsequent drug entities utilized standard curve concentrations of 25, 37.5, 50, 62.5, and 75 mcg/mL. Precision was evaluated from 10 replicate injections of fentanyl 10 mcg/mL and 25 mcg/mL, hydromorphone 50 mcg/mL, ketamine 50 mcg/mL, midazolam 40 and 50 mcg/mL, morphine 50 mcg/mL, and pentobarbital 50 mcg/mL. Each drug entity analyzed was subjected to forced degradation with 1N hydrochloric acid, 1N sodium hydroxide, 3% hydrogen peroxide, and heat. 

Four 5 mL samples of each drug and concentration listed in Table 2 were prepared in 10 mL polypropylene syringes and sealed with syringe tip caps. Fentanyl 50 mcg/mL, ketamine 10 mg/mL, midazolam 5 mg/mL, and pentobarbital 50 mg/mL samples did not require further dilution. Fentanyl 10 mcg/mL in 0.9% sodium chloride samples were compounded from fentanyl 50 mcg/mL. Hydromorphone 100 mcg/mL in 0.9% sodium chloride samples were compounded from hydromorphone 2 mg/mL. Midazolam 0.4 mg/mL in 5% dextrose samples were compounded from midazolam 5 mg/mL. Morphine 1 mg/mL in 0.9% sodium chloride and morphine 1 mg/mL in 5% dextrose samples were compounded from morphine 50 mg/mL. Triplicate determinations of the four samples were obtained initially, on day 50 or 51, and on day 100. The samples were stored at ambient room temperature (~20 °C–25 °C) in a locked cabinet, without the presence of light. The samples were defined as being stable if the concentration at the time of analysis was > 90% of the original analysis. Triplicate determinations of the corresponding duplicate quality control samples (fentanyl 10 mcg/mL, fentanyl 25 mcg/mL, hydromorphone 50 mcg/mL, ketamine 50 mcg/mL, midazolam 50 mcg/mL, morphine 50 mcg/mL, pentobarbital 50 mcg/mL) were performed on each day of analysis for the compound of interest. Blank injections were systematically included in the analyses.

Physical assessment. After 100 days of storage, all samples were placed in borosilicate colorless glass test tubes and visually inspected with the aid of white and black backgrounds for clarity, color changes, and particulate matter.

## 3. Results and Discussion

With the exception of the hydromorphone 100 mcg/mL dilution, all compounds were found to contain greater than 95% of their initial concentration remaining at 100 days (Table 2). Retention times of the analytes of interest and degradants are listed in Table 2 along with linearity, precision, intraday coefficients of variation, and inter-day coefficients of variation validation data. Degradation peaks did not interfere with the corresponding analyte of interest for any of the drug compounds. All standard curves had a linearity of > 0.999. Precision analysis from 10 replicate injections of each analyte of interest resulted in a percent relative standard deviation of < 1. Quality control samples for each compound had an intraday coefficient of variation < 1.5% and an interday coefficient of variation < 3%. When visually inspected in borosilicate colorless glass test tubes with the aid of white and black backgrounds all samples were found to be clear and colorless after 100 days of storage.

The study results indicate that all analyzed compounds of fentanyl, ketamine, midazolam, morphine, and pentobarbital are stable for 100 days when stored at room temperature in polypropylene syringes. The hydromorphone 100 mcg/mL in 0.9% sodium chloride compound was found to have 90.3% of its original concentration remaining at 100 days. It must be noted that the standard deviation for this compound was 0.7%, thereby indicating the probability that some samples stored for 100 days would be below the defined 90% cutoff and therefore be designated as unstable for the stated time period. USP Chapter <797> standards for compounded sterile products do not allow for room temperature storage of compounded products beyond 48 hours for low-risk level compounded sterile preparations (CSPs), 30 hours for medium-risk level CSPs, and 24 hours for high-risk level CSPs, in the absence of passing sterility tests [7]. The results presented herein are for stability only and do not imply sterility for the storage durations.

## 4. Conclusions

Fentanyl 10 mcg/mL in 0.9% sodium chloride, fentanyl 10 mcg/mL in 5% dextrose, fentanyl 50 mcg/mL, ketamine 10 mg/mL, midazolam 0.4 mg/mL in 5% dextrose, midazolam 5 mg/mL, morphine 1 mg/mL in 0.9% sodium chloride, morphine 1 mg/mL in 5% dextrose, and pentobarbital 50 mg/mL are stable for at least 100 days when stored at room temperature in polypropylene syringes. Hydromorphone 100 mcg/mL in 0.9% sodium chloride is stable for at least 50 days when stored at room temperature in polypropylene syringes. 
